# Assessment of sorafenib induced changes in tumor perfusion of uveal melanoma metastases with dynamic contrast-enhanced ultrasound (DCE-US)

**DOI:** 10.1007/s00432-021-03666-8

**Published:** 2021-05-28

**Authors:** Dane Wildner, Lucie Heinzerling, Max E. Scheulen, Eckhart Kaempgen, Gerold Schuler, Deike Strobel, Rolf Janka, Markus F. Neurath, Joerg Sturm, Ferdinand Knieling

**Affiliations:** 1 Division of Ultrasound, Department of Internal Medicine 1, University Hospital Erlangen, Friedrich-Alexander-University Erlangen-Nuremberg, Ulmenweg 18, 91054 Erlangen, Germany; 2 Department of Dermatology, University Hospital Erlangen, Friedrich-Alexander-University Erlangen-Nuremberg, Ulmenweg 18, 91054 Erlangen, Germany; 3 Department of Internal Medicine (Tumor Research), University Hospital Essen, Hufelandstr. 55, 45147 Essen, Germany; 4 Radiology Institute, University Hospital Erlangen, Maximiliansplatz 1, 91054 Erlangen, Germany; 5 Department of Pediatrics and Adolescent Medicine, University Hospital Erlangen, Friedrich-Alexander-University Erlangen-Nuremberg, Loschgestr. 15, 91054 Erlangen, Germany

**Keywords:** Dynamic contrast-enhanced ultrasound (DCEUS), Sorafenib, Uveal melanoma, Functional imaging, CEUS, Response prediction

## Abstract

**Purpose:**

Dynamic contrast-enhanced ultrasound (DCE-US) was used to monitor early response to sorafenib therapy in patients with liver metastases from uveal melanoma.

**Methods:**

In total, 21 patients with liver metastases were recruited within a prospective trial and underwent daily sorafenib therapy. DCE-US of a target lesion was performed before initiation of treatment, on day 15 and 56. Two independent blinded investigators performed software analysis for DCE-US parameters and inter-observer-correlation was calculated. Response to treatment was evaluated on day 56. DCE-US parameters were correlated with clinical response and RECIST1.1 criteria.

**Results:**

Inter-observer-correlation (*r*) of DCE-US parameters [time-to-peak (TTP), mean-transit-time (MTT), peak intensity (PI), regional blood volume (RBV), regional blood flow (RBF)] at baseline, day 15, and day 56 was highly significant (*r*-range 0.73–0.97, all *p* < 0.001). Out of 17 evaluable patients, 12 patients survived day 56 (clinical responders, cRE), whereas, five patients died before day 56 and were classified as non-responders (cNR). TTP values significantly increased in the cRE group 15 days after initiation of treatment for investigator 1 (*p* = 0.034) and at day 56 for both investigators (*p* = 0.028/0.028). MTT had increased significantly in the cRE group on day 56 (*p* = 0.037/0.022). In the cNR group changes for TTP and MTT remained insignificant. Thus, increase of the DCE-US parameters TTP and MTT are associated with response to treatment and prognosis.

**Conclusion:**

An increase of TTP and MTT at frequent intervals could serve as a surrogate marker for early response evaluation to anti-angiogenic treatment of metastatic uveal melanoma.

**Supplementary Information:**

The online version contains supplementary material available at 10.1007/s00432-021-03666-8.

## Introduction

Uveal melanoma is the most frequent primary intraocular malignant tumor in adults (Chattopadhyay et al. 2016). In the last decades, treatment for patients with cutaneous melanoma has significantly improved, but this progress could not be transferred to patients with uveal melanoma. Although we know that cutaneous and uveal melanocytes both derive from neural crest cell migration, their malignant potential and pathologic behavior is different (Luke et al. 2013).

Thus, uveal melanoma remains a tumor entity with limited therapy options and for which effective treatment approaches are lacking (Carvajal et al. 2017). Even though sorafenib did not show convincing effectiveness in metastatic cutaneous melanoma, an effect was postulated in uveal melanoma known for the high vascularization of metastases. Therefore, clinical trials evaluated the efficacy of sorafenib in the treatment of patients with metastatic uveal melanoma (Carvajal et al. 2017; Mouriaux et al. 2016; Scheulen et al. 2017).

Furthermore, early assessment of response is becoming more and more important with a number of approaches currently being investigated (Morin et al. 2018). For anti-angiogenic or immunotherapies structural changes of the tumor tissue might be present before a decrease of tumor size occurs. Therefore, it would be desirable to complement the established criteria of oncological imaging (i.e. response evaluation criteria in solid tumors—RECIST) (Hodi et al. 2016). Specifically, novel techniques for functional imaging are the focus of current research (Lassau et al. 2014; Lambin et al. 2017; Blomley and Eckersley 2002; Carter et al. 2018).

Because it is broadly available, radiation-free and cost-effective the use of ultrasound is widely recommended and performed during the initial diagnostic investigation and in the follow-up of patients with uveal melanoma (Nathan et al. 2015; Choudhary et al. 2016). As the application of ultrasound contrast agents (UCA) was established in the characterization of suspicious focal liver lesions (Claudon et al. 2013), contrast-enhanced ultrasound (CEUS) is increasingly being used for non-hepatic applications such as ocular lesions (Li et al. 2018; Sidhu et al. 2018).

Relating to functional imaging, CEUS offers the unique opportunity to visualize tumor perfusion safely, in vivo and in real-time. Dynamic contrast-enhanced ultrasound (DCE-US) represents the quantitative analysis of UCA-kinetics, displayed by a Time-intensity-curve, with a high-temporal resolution (Dietrich et al. 2012). The perfusion kinetics of UCA in tumor tissue can be analyzed before and after antiangiogenic therapy at frequent intervals. Up to date an increasing number of publications address the potential of DCE-US in oncology (Lassau et al. 2014; Amadori et al. 2018; Chami et al. 2011; Knieling et al. 2013; Mogensen et al. 2017).

Data suggest this as a feasible clinical approach to monitor individual response to therapy in patients with hepatic metastases of uveal melanoma during sorafenib treatment. The aim of this study was to evaluate the ability of DCE-US to predict response to treatment early in the course of treatment.

## Materials and methods

### Study design

An institutional review board approved the randomized, double-blinded, placebo-controlled prospective multicentric phase II study of sorafenib in patients with chemonaive metastatic uveal melanoma (ClinicalTrials.gov Identifier: NCT01377025). The study was conducted at three university hospitals. The primary outcome measure was progression-free survival (results of the original study are not part of this manuscript). DCE-US evaluation could be performed optionally as a secondary outcome measure, if available at the participating center. During the trial, DCE-US measurements were carried out only at our study site. All procedures performed in studies involving human participants were in accordance with the ethical standards of the institutional ethical committee (Ethical Committee, Friedrich-Alexander-University Erlangen-Nuremberg; Reference Number 40_13 B) and with the 1964 Helsinki declaration and its later amendments or comparable ethical standards. Informed consent was obtained from all individual participants included in the study after thorough patient information. In a first part of the study all patients received an oral daily dose of 400 mg sorafenib (run-in phase: from initiation of sorafenib therapy up to day 56). Treatment was continued until progression or unacceptable toxicity occurred. After the run-in phase patients were randomized into a treatment-group and a placebo-group. DCE-US was performed only in the run-in phase, in each patient on days 0, 15 and 56. Initial staging and re-staging were done before the start of treatment and at the end of the run-in phase according to RECIST 1.1 criteria. In this part of the study, evaluation of response was defined, according to RECIST, as a progression with a 20% increase in the sum of the longest diameters of target lesions or the detection of new lesions in MRI on day 56.

### Patient population

Patients were suitable for study inclusion if they met the following inclusion criteria: at least 18 years of age, histologically or cytologically proven metastatic uveal melanoma with confirmation of liver metastases, at least one unidimensional measurable lesion ≥ 10 mm, chemonaive patient with eligibility for sorafenib treatment in the study.

### Contrast-enhanced ultrasound (CEUS) with perfusion quantification (DCE-US)

**Fig. 1:**
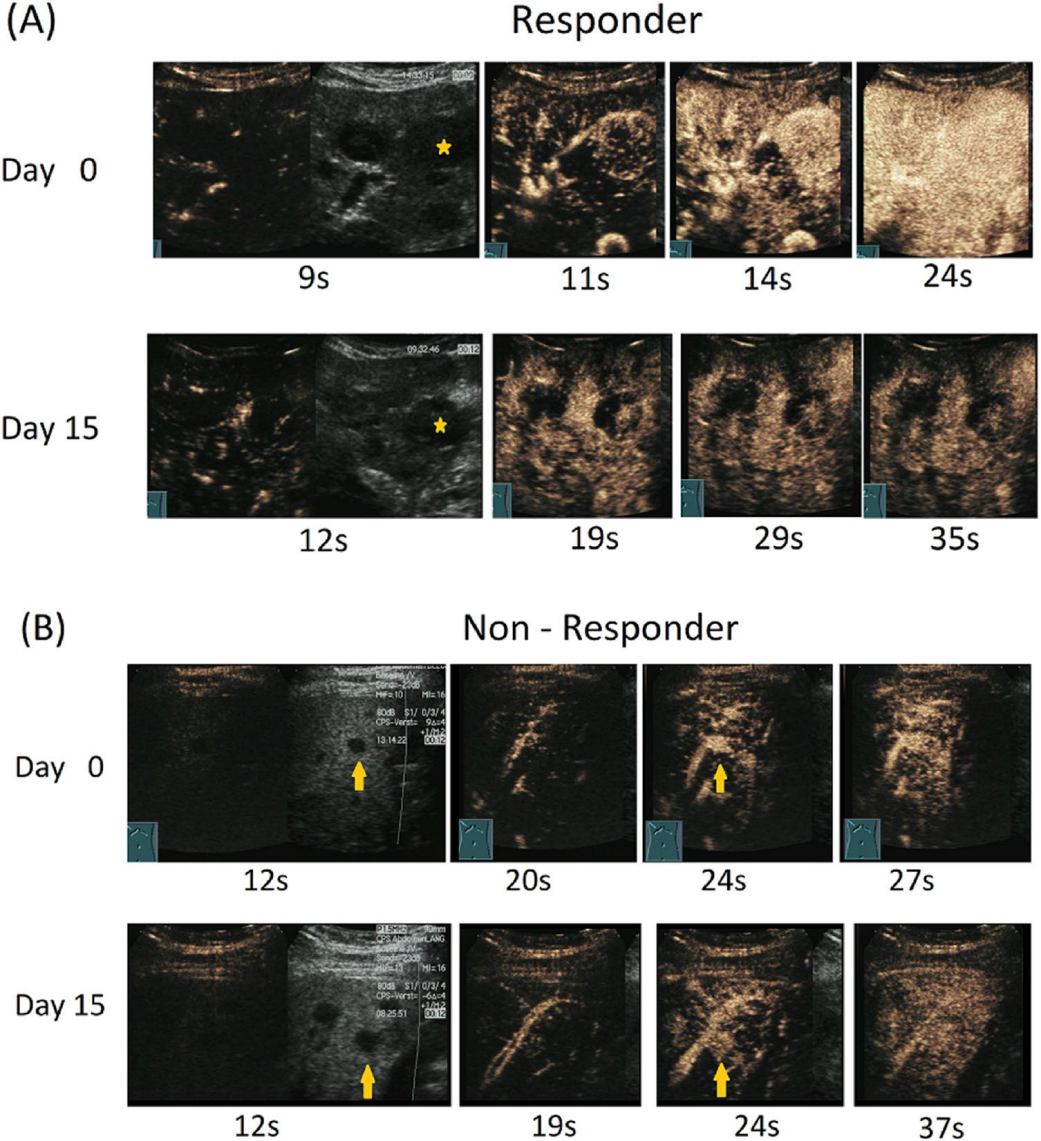
Contrast-enhanced ultrasound examinations: The first image showing the dual window mode with the lesion in grey scale ultrasonography and at the beginning of contrast agent wash-in. Image series of two patients after initiation of sorafenib therapy at baseline (d0) and after 2 weeks of treatment (d15). **a** Patient with response to sorafenib treatment. A decline of signal intensity within the tumor and the occurrence of avascular areas (remaining black) in the lesion (*) are visible. The tumor size is unchanged. **b** Patient without response to treatment. The lesion (↑) remains well vascularized throughout the arterial wash-in phase. Again, the lesion size is constant

CEUS examinations were performed according to EFSUMB guidelines and recommendations (Claudon et al. 2013). Two experienced sonographers, performed CEUS examinations using a Siemens Sequoia 512 ultrasound machine equipped with a curved array probe (4C1 probe). Initially a baseline B-mode examination was performed to assess the targeted lesion and largest diameter. In case of more than one metastasis, a single target lesion was selected based on size and site (e.g. good acoustic window) for all further DCE-US examinations. For contrast signal acquisition, the preset was changed (low mechanical index, dual window mode). Then, the UCA SonoVue^®^ (equates to Lumason^®^, Bracco, Italy) was reconstituted according to manufacturer’s instructions. A bolus of 1.2 ml UCA was administered followed by a 10 ml saline flush through a cubital vein. As needed, the patients were advised to hold breath during scanning. The arterial phase of UCA wash-in was recorded (up to 45 s) and stored as video files for following quantitative software analysis (Fig. 1).

### Quantitative DCE-US analysis

**Fig. 2:**
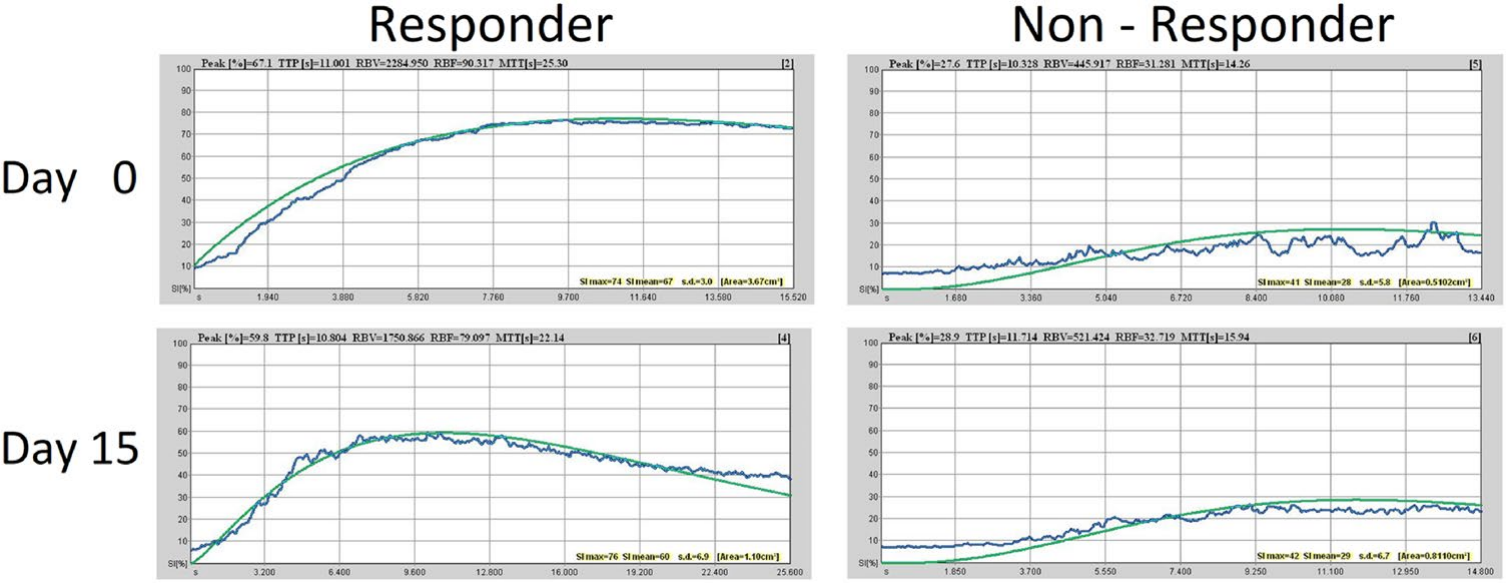
Time-intensity-curves of two patients, responder and non-responder, at baseline (Day 0) and after two weeks of sorafenib therapy (Day 15). Blue line showing the raw image data of absolute signal intensity. On that basis, the green line (fitted curve) is calculated and DCE-US parameters are obtained

The video files were transferred from the ultrasound machine to a separate workstation. Two independent examiners, both blinded to clinical course and CEUS examinations performed the quantification. All videos were analyzed with Qontrast^®^ software (Esaote S. p. A., Italy). For each investigation, a color-coded map with corresponding flow parameters was calculated to describe the characteristics of UCA behavior during DCE-US investigation. The amount of microbubbles is proportional to the signal intensity derived from CEUS. These microbubbles circulate strictly intravasal, the characteristic flow parameters were related to the vascularization of the region of interest (ROI). DCE-US parameters provide a relative, semi-quantitative evaluation of physiologic parameters, like for example blood flow and blood volume based on the dye-dilution theory (Dietrich et al. 2012). Peak enhancement (PE) represented the difference between the maximum amplitude (*I*_max_) and baseline (*I*_0_) and is proportional to the UCA dose, and therefore, an indicator for relative blood volume in the corresponding ROI. Time-to-peak (TTP) was the period required for UCA to arrive in the ROI and reach PE. The values $A$, $\beta$, and $A\beta$ were used to represent relative blood volume (RBV), flow velocity or mean transit time (MTT) and relative blood flow (RBF). The ROI was placed in the remaining vascularized vital tumor tissue as confirmed by color-coded DCE-US maps. Areas of necrosis were excluded from analysis. For subsequent analysis, the same ROI was chosen at each time point. The videos had to have sufficient video quality for eligibility (Fig. 2).

### Response evaluation

MRI- (Magnetom^®^ Avanto, Siemens Healthcare Diagnostics GmbH, Eschborn, Germany. Contrast agent: Gadovist^®^ Bayer Vital GmbH Leverkusen, Germany) scans were scheduled before start of sorafenib treatment and after 2 months of anti-angiogenic treatment. Patients were categorized as responders or non-responders to treatment according to Response Evaluation Criteria in Solid Tumors, Version 1.1 (RECIST 1.1) (Eisenhauer et al. 2009) by an independent expert radiologist. The international RECIST 1.1 classification is based on the changes from baseline in the sum of the longest tumor diameter. Four response categories can be distinguished: complete remission (complete disappearance of tumor lesions) [CR], partial remission (at least 30% decrease of sum of longest tumor diameters) [PR], stable disease (between 30% decrease and 20% increase in sum) [SD] and progressive disease (more than 30% increase in sum) [PD]. CR, PR and SD according to RECIST 1.1 were subsumed as responders (RE) and PD was defined as non-responders (NR) to treatment. Progression-free survival and overall survival were defined as time from treatment initiation to appearance of PD on MRI scans and time from treatment initiation to death, respectively. The radiologist was blinded to the results of DCE-US.

A second clinical response evaluation was conducted to compare survival. Patients surviving day 56 of treatment were considered clinical responders (cRE) meanwhile patients who died within two months of treatment were considered clinical non-responders (cNR).

### Statistical analysis

Distributions of demographic and clinical data were expressed as means ± standard deviation. All values of different DCE-US flow parameters were calculated for both investigators and separately between day 0, 15 and day 56 for each subpopulation of patients. Spearman’s correlation of DCE-US quantification values between both observers for days 0, 15 and 56 was performed to assess the inter-observer-correlation.

Non-parametric Wilcoxon test was used to compare DCE-US parameters of the subgroup of patients with MRI reevaluation via RECIST1.1 at day 56 as well as for the comparison of DCE-US values of cRE and cNR based on survival at day 56. Again, calculation was performed separately for both examiners at each time point.

Statistical analysis was conducted using SPSS 19 (version 19.0.0.1, IBM SPSS statistics, Armonk, New York, USA) GraphPad Prism version 6.00 (GraphPad Software, La Jolla, CA, USA) and Microsoft Excel Software. All reported *p* values are two-sided and *p* value < 0.05 was considered statistically significant.

## Results

### Patient characteristics

In total, 21 patients were included in the study. Out of these, three patients had to be excluded because of video capturing errors (*n* = 2) and video storage error on day 15 (*n* = 1). Another patient had to terminate the study medication due to an intolerance of sorafenib before day 56 and could not be included. Patients’ characteristics are displayed in Table 1.

**Table 1:** Patient characteristics

Patient number	Age (years)	Gender (male/female)	Size of liver metastasis (mm)	Hb (g/dl)	ALT (U/l)	Albumin (g/dl)	LDH (U/l)	S100 (μg/l)	MIA (ng/ml)	ΔRECIST d56-d0 (sum mm/%)	cRE	cNR
1	66	F	23	13.9	19	47.4	237	0.04	–	1/− 4.2%	X	
2	50	M	20	14.7	47	43.9	324	0.19	53.3	–	X	
3	65	F	30	11.8	24	41.9	298	0.10	12.3	0/0%	X	
4	64	M	38	14.2	23	39.5	610	14.00	63.8	7/− 5.3%	X	
5	53	M	93	12.1	60	40.4	1461	0.72	192.4	†		X
6	79	M	21	12.3	13	45.5	240	0.06	5.9	–	X	
7	70	M	33	11.4	80	34.7	741	0.08	11.9	†		X
8	72	M	17	15.5	39	43.2	606	0.36	43.1	†		X
9	71	M	74	12.7	44	–	222	0.05	30.1	–	X	
10	54	M	40	13.3	35	39.5	1048	0.11	35.8	†		X
11	44	F	38	14.9	157	47.6	732	0.06	12.8	− 8/21.1%	X	
12	49	M	12	14.1	70	49.1	438	0.08	53.8	6/17.6%	X	
13	57	F	13	15.2	50	46.9	296	0.06	7.7	− 1/2.4%	X	
14	55	M	15	13.7	50	36.6	495	0.59	16.2	†		X
15	53	F	23	15.3	16	44.0	339	0.10	6.0	1/− 6.7%	X	
16	52	M	34	14.9	15	48.0	188	0.06	3.9	6/− 11.1%	X	
17	47	F	14	13.6	84	42.3	287	0.06	3.9	6/− 13.0%	X	
Total (M ± SD)	58.9 ± 10.2	♂: 11 (65%) ♀: 6 (35%)	31.6 ± 21.9	13.7 ± 1.3	48.6 ± 35.8	43.2 ± 4.2	503.6 ± 340.8	1.0 ± 3.4	34,6 ± 46,6		*n* = 12	*n* = 5

Laboratory values at day 0: *Hb* haemoglobin, *ALT* alanine aminotransferase, *LDH* lactate dehydrogenase, *S100* calcium binding Protein S100, *MIA* melanoma inhibitory activity, *RECIST* response evaluation criteria in solid tumors—Version 1.1, *cRE* clinical responder to sorafenib therapy at day56, *cNR* clinical non-responder to sorafenib therapy at day 56, *M* mean, ± *SD* standard deviation

^†^Patient died before day 56

Two different investigators analyzed the videos of the remaining 17 patients using Qontrast^®^ quantification software. Due to patients’ death and video quality requirements, the final analysis in the sorafenib treatment group included 44 videos, 17 at baseline, 17 at day 15 and 10 at day 56. Seven videos could not be obtained or analyzed on day 56 because of patients’ death (﻿† *n* = 5), withdrawal of therapy due to adverse effects (*n* = 1) or bad video quality (*n* = 1).

### Inter-observer correlation of DCE-US parameters

Inter-observer correlation of DCE-US parameters (TTP, MTT, Peak, enhancement, RBV and RBF) showed good accordance. The comparison of the parameters at each time point showed a good correlation [Spearman correlation coefficient *r* at baseline day 0: 0.86 for MTT; 0.96 for Peak intensity; 0.92 for RBV; 0.96 for RBF. *r* at day 15: 0.95 for TTP; 0.81 for MTT. *r* at day 56: 0.94 for MTT; 0.96 for Peak intensity; 0.94 for RBV; 0.95 for RBF with *p* < 0.0001 for all of these values]. Analysis of DCE-US parameters for both investigators at different time points are shown in Table 2.

**Table 2:** Intraclass correlation [Spearman correlation coefficient *r*] of all 44 DCE-US parameters performed by Investigator 1 and 2 (M ± SD: means ± standard deviation) at different time points, showing good correlations of the results

DCE-US parameter	Investigator 1	Investigator 2	*r*	95%CI	*p* value
Day 0	*n* = 17	*n* = 17			
TTP [s]	12.1 ± 2.3	12.1 ± 2.3	0.75	0.41–0.90	0.0005
MTT [s]	21.3 ± 4.8	21.4 ± 5.7	0.86	0.66–0.95	< 0.0001
Peak [%]	52.7 ± 14.9	54.1 ± 14.5	0.96	0.88–0.98	< 0.0001
RBV [a.u.]	1502.1 ± 799.0	1491.5 ± 727.3	0.92	0.79–0.97	< 0.0001
RBF [a.u.]	66.2 ± 21.9	67.6 ± 21.8	0.96	0.90–0.99	< 0.0001
Day 15	*n* = 17	*n* = 17			
TTP [s]	14.8 ± 4.0	14.5 ± 3.8	0.95	0.85–0.98	< 0.0001
MTT [s]	23.2 ± 7.0	23.6 ± 8.3	0.81	0.54–0.93	< 0.0001
Peak [%]	44.5 ± 10.5	44.5 ± 9.5	0.79	0.49–0.92	0.0002
RBV [a.u.]	1262.3 ± 590.5	1317.7 ± 663.7	0.73	0.38–0.90	0.0009
RBF [a.u.]	53.5 ± 16.3	53.8 ± 14.3	0.75	0.43–0.91	0.0005
Day 56	*n* = 10	*n* = 10			
TTP [s]	16.7 ± 3.8	17.0 ± 4.3	0.80	0.34–0.95	0.0055
MTT [s]	28.4 ± 11.5	30.3 ± 12.2	0.97	0.86–0.99	< 0.0001
Peak [%]	36.1 ± 17.0	36.6 ± 16.7	0.96	0.83–0.99	< 0.0001
RBV [a.u.]	1126.6 ± 671.2	1177.9 ± 607.4	0.94	0.77–0.99	< 0.0001
RBF [a.u.]	41.8 ± 20.2	42.4 ± 19.5	0.95	0.81–0.99	< 0.0001
All values	*n* = 44	*n* = 44			
TTP [s]	14.2 ± 3.8	14.1 ± 3.8	0.89	0.81–0.94	< 0.0001
MTT [s]	23.6 ± 7.9	24.3 ± 9.0	0.91	0.83–0.95	< 0.0001
Peak [%]	45.8 ± 15.0	46.4 ± 14.7	0.94	0.89–0.96	< 0.0001
RBV [a.u.]	1324.1 ± 696.2	13,531.1 ± 673.2	0.86	0.76–0.92	< 0.0001
RBF [a.u.]	55.8 ± 21.3	56.6 ± 20.8	0.93	0.87–0.96	< 0.0001

### Clinical response evaluation

Clinical response to sorafenib treatment was evaluated in 17 patients on the basis of survival at day 56. Patients were classified as clinical responders (cRE) to treatment if surviving day 56 and as clinical non-responders (cNR) if they did not reach this point in time. 12 patients were classified cRE, five patients were classified cNR. B-mode diameter of the target lesion showed an decrease from 31.65 ± 21.87 mm to 31.00 ± 21.73 mm (*p* = 0.52) at day 15 and 27.18 ± 14.59 mm (*p* = 0.0468) at day 56. The subanalyses for cNR and cRE did not show any significant differences (data not shown).

DCE-US parameters of cRE and cNR are presented in Table 3. Baseline flow parameters showed no significant difference between cRE and cNR group. In cNR group no significant difference for all DCE-US values was found between baseline and day 15.

**Table 3:** Clinical non-responders (cNR) and responders (cRE)

DCE-US parameter	Investigator 1			Investigator 2		
	Baseline–day 0	Day 15	Day 56	Baseline–day 0	Day 15	Day 56
cNR						
TTP [s]	11.2 ± 1.8	13.2 ± 2.7	n/a	10.8 ± 1.0	12.8 ± 3.4	n/a
MTT [s]	22.1 ± 5.5	25.0 ± 10.3	n/a	21.2 ± 6.2	22.8 ± 13.0	n/a
Peak [%]	58.0 ± 18.1	47.8 ± 11.5	n/a	60.3 ± 13.4	45.1 ± 5.6	n/a
RBV [a.u.]	1923.9 ± 1092.1	1567.8 ± 855.4	n/a	1706.6 ± 873.5	1315.4 ± 984.8	n/a
RBF [a.u.]	75.1 ± 25.7	60.1 ± 17.3	n/a	76.1 ± 23.0	54.75 ± 9.6	n/a
cRE						
TTP [s]	12.5 ± 2.4	15.4 ± 4.4^a^	16.7 ± 3.8^d^	12.7 ± 2.5	15.2 ± 3.8	16.9 ± 4.3^e^
MTT [s]	20.9 ± 4.8	22.5 ± 5.7	28.4 ± 11.5^f^	21.5 ± 5.8	23.9 ± 6.3	30.3 ± 12.2^g^
Peak [%]	50.4 ± 13.7	43.2 ± 10.3^b^	36.14 ± 17.0	51.6 ± 14.6	44.2 ± 10.9	36.55 ± 16.7
RBV [a.u.]	1326.4 ± 616.6	1135.0 ± 425.4	1126.6 ± 671.2	1402.0 ± 680.0	1318.6 ± 536.7	1177.93 ± 607.4
RBF [a.u.]	62.5 ± 20.2	50.8 ± 15.7^c^	41.8 ± 20.2	64.1 ± 21.3	53.5 ± 16.3	42.4 ± 19.5

DCE-US parameter of the cNR-group showed no significant differences at day 15 compared to baseline values at all. In the cNR-group all patients had died before day 56 and no DCEUS-parameters could be obtained (n/a: not applicable)

In the cRE-group we found significant differences in DCE-US values compared to baseline for: day 15 vs baseline:

^a^TTP, *p* = 0.034

^b^PE, *p* = 0.050

^c^RBF, *p* = 0.041 and for day 56 vs. baseline

^d^TTP, *p* = 0.028

^e^TTP, *p* = 0.028

^f^MTT, *p* = 0.037

^g^MTT, *p* = 0.022

**Fig. 3:**
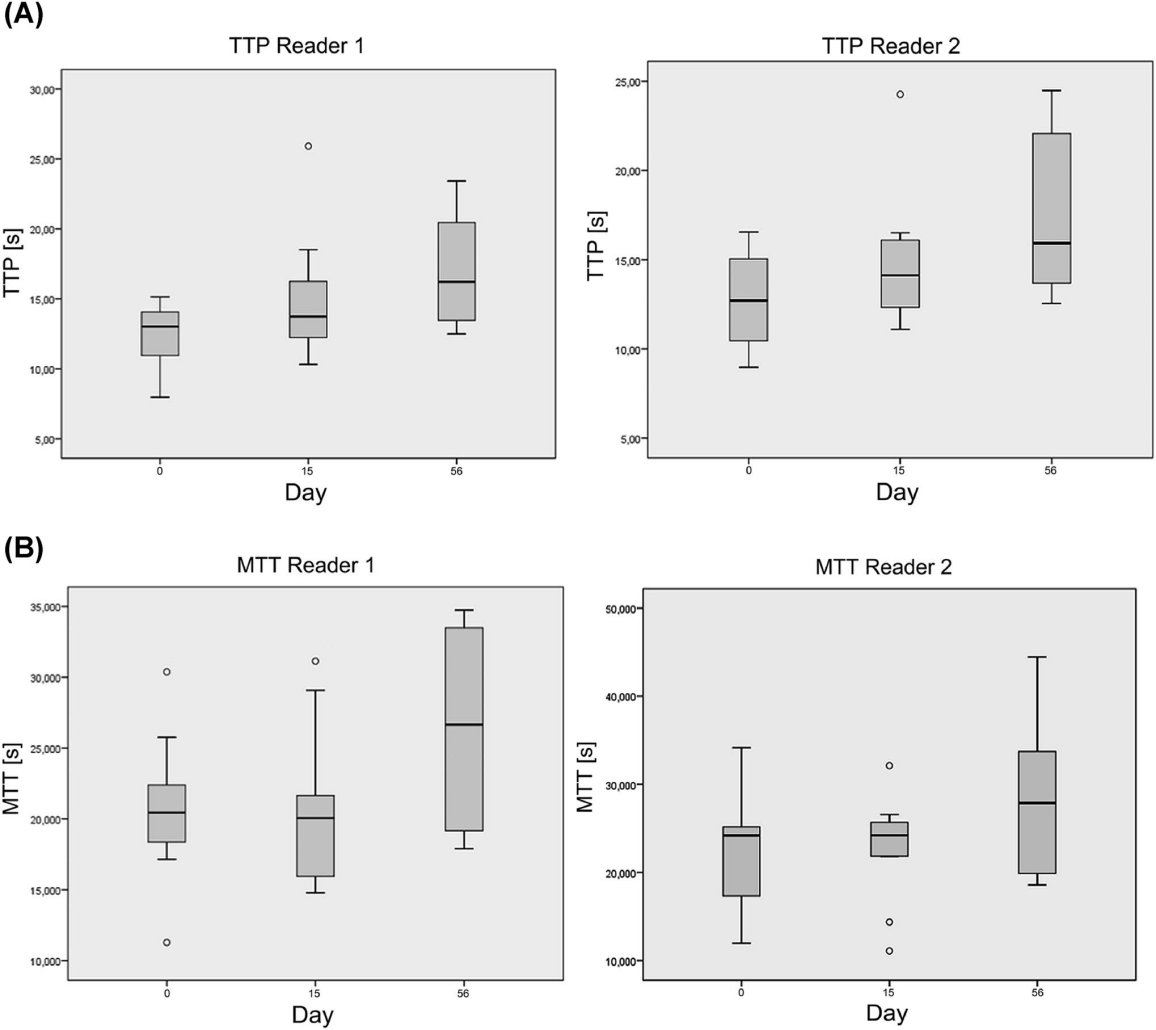
**a** Time-to-peak (TTP) and **b** Mean-transit-time (MTT) values of both readers at baseline, day 15 and day 56. Each box represents the interquartile range and the median. Error bars mark minimum and maximum values (range) and small circles and stars mark outliers

In contrast, cRE showed significant differences in DCE-US values. A statistically significant increase of TTP (*p* = 0.034), Peak Intensity (*p* = 0.05) and RBF (*p* = 0.041) was observed for investigator 1 at day 15. The comparison of TTP (*p* = 0.099), Peak Intensity (*p* = 0.084) and RBF (*p* = 0.060) of investigator 2 just fell short of being statistically significant. MTT and RBV showed no significant difference for both investigators at day 15. TTP and MTT were significantly increased at day 56 compared to baseline (Fig. 3). Peak intensity, RBV and RBF did not change significantly.

### Radiological response evaluation (RECIST 1.1)

Radiological response to sorafenib treatment was evaluated according to RECIST 1.1 criteria at baseline and after 2 months. Patients in the sorafenib group were classified as responders (RE) [including complete remission (CR), partial remission (PR), and stable disease (SD)] or non-responders (NR) for statistical analysis. Out of 17 consecutive patients five patients died during the sorafenib treatment period of 2 months and therefore could not be reevaluated. In another three patients the RECIST evaluation was not possible because the patients refused the MRI-examination at day 56. For the final radiological analysis nine patients were included in the per protocol evaluation by RECIST 1.1 criteria. All of these patients were from the cRE-group. According to RECIST 1.1 protocol, the mean RECIST-sum (mm) was 44.3 ± 33.2 at baseline and 46.3 ± 33.2 at day 56, with a mean change of the RECIST-sum by 2 ± 4.6, showing no statistical significance (*p* = 0.26). Three patients had an increase, one patient presented with stable tumor size and five patients had a decrease in tumor size (Table 1).

Concerning DCE-US parameters of these patients, there was no statistically significant difference between baseline and day 15 for both readers. The comparison of TTP at baseline and day 56 showed a significant difference (*p* = 0.036) for investigator 1. TTP values of investigator 2 just fell short of being statistically significant (*p* = 0.069). For MTT both investigators found a significant increase of values from baseline (20.78 ± 5.35/22.02 ± 6.65) compared to day 56 (26.41 ± 7.45/28.25 ± 9.09) (*p* = 0.050/0.036). Peak Intensity, RBV and RBF failed statistical significance over the treatment period.

## Discussion

In this study, we examined quantitative CEUS to recognize early changes of tumor perfusion during sorafenib therapy in patients with metastatic uveal melanoma. This is of critical relevance in the setting of potentially rapid tumor progression during anti-cancer treatment. DCE-US is able to demonstrate perfusion changes within a short period of antiangiogenic treatment. The application of CEUS is not limited by radiation exposure and can be repeated safely and easily at short intervals. A significant decrease of tumor perfusion could be observed already after 15 days of sorafenib treatment, represented by extension of DCE-US parameters for blood flow (TTP, MTT). In addition, parameters correlating with the circulating blood volume (PE, RBV) were also decreasing (Table 3).

Since neovascularization is one of the major events occurring during tumor growth, anti-angiogenic treatment is explored in many entities—so also in uveal melanoma. However, the number of patients profiting from such regimens is low (Mouriaux et al. 2016; Carvajal et al. 2017). One major challenge is how to identify patients with benefit from such a therapy and to identify individual therapeutic response as early as possible.

Classical approaches measuring a therapeutic response include clinical and radiological findings. In the past, strict and well-defined response criteria (RECIST) using CT or MRI (Eisenhauer et al. 2009), have been established in oncological protocols. Measurements are based on the change of extent or diameter of target lesions during the course of therapy. Especially with the new therapeutic regimens, the initial reduction in tumor size, measured by RECIST, has been reported to be very low (Llovet et al. 2008; Shepherd et al. 2005). We could observe similar findings in our study: some patients, who were held responders according to RECIST 1.1 criteria, even showed a slight increase in total RECIST sum. Therefore, novel imaging techniques with the capability of real time morphological (size) and functional (perfusion) visualization are gaining increased attention. In this regard, ultrasound contrast agents enable continuous visualization of the blood flow inside a target lesion. Post processing of the acquired data can give insight into specific vascular changes (Knieling et al. 2013; Lassau et al. 2010, 2011a, b). Furthermore, DCE-US offers many advantages over imaging techniques like CT or MRI (e.g. higher temporal resolution, cost effectiveness, excellent tolerance and safety profile of UCA) (Claudon et al. 2013).

In this study, we found a strong correlation between DCE-US parameters of two blinded readers, suggesting a very good reproducibility. These results were substantiated by findings of Ridolfi et al. (2012) who observed nearly perfect agreement (MTT: *κ* = 0.87; TTP: *κ* = 0.90) for DCE-US parameters with Qontrast^®^ software in liver application.

The presented data shows significant differences between several DCE-US parameters in clinical and radiological RE measured between day 0 and 56. Furthermore, with regard to survival, differences between cRE and cNR group can be found as early as 15 days after initiation of anti-angiogenic treatment. However, significance levels of both readers were inconsistent due to the small sample size. Lassau et al. (2011a, b) demonstrated that several DCE-US parameters measured between day 0 and 3 of anti-angiogenic treatment in HCC treatment indicated satisfying correlation with early tumor response (AUC: *p* = 0.02; TTP: *p* = 0.03). Comparable results could be seen in patients with HCC treated with sorafenib (Knieling et al. 2013), renal cell carcinoma treated with sunitinib (Lassau et al. 2010) or breast cancer patients under neoadjuvant chemotherapy (Saracco et al. 2017) for instance.

Main limitations of the present study include, incoherence concerning some of the calculated changes of DCE-US parameters due to small sample size and the only modest effect of sorafenib on angiogenesis that lowered the discrepancy between responders and non-responders (Mouriaux et al. 2016). Furthermore, two-dimensional US images in a single plane may not be representative for the targeted lesion as a whole. In addition, the exclusion of necrotic areas during the quantification process will not reveal significant changes in several perfusion parameters such as relative blood volume (RBV), relative blood flow (RBF), and maximum peak enhancement (Peak). Consequently, the development of 3D perfusion techniques should be the next step (Dong et al. 2016).

Given its radiation-free character and good interrater reproducibility, quantitative DCE-US might be a promising tool being implemented in the evaluation of novel anti-angiogenic treatment strategies.

## Conclusion

DCE-US is a cost-effective, safe and repeatable technique, which can reliably detect early changes in tumor perfusion. Combined with software quantification, it can be used to identify individual therapeutic response early in the course of anti-angiogenic treatment. Further DCE-US studies evaluating the clinical relevance for response prediction should be performed in the context of oncological clinical trials.

## Supplementary Information

Below is the link to the electronic supplementary material.

Supplementary file1 ESM_1 (Video file) Clinical Non-Responders (cNR) and Responders (cRE) (MP4 69500 kb)

## Abbreviations

ALT: Alanine aminotransferase
AST: Aspartate aminotransferase
AUC: Area under the curve
AVI: Audio video interleave
CAD: Coronary artery disease
cNR: Clinical non-responder
CR: Complete remission
cRE: Clinical responder
DCE-US: Dynamic contrast-enhanced ultrasound
DEGUM: Deutsche Gesellschaft für Ultraschall in der Medizin (German Society for Ultrasound in Medicine)
ECG: Electrocardiography
ECOG: Eastern cooperative oncology group
EFSUMB: European federation of societies for ultrasound in medicine and biology
Hb: Haemoglobin
HCC: Hepatocellular carcinoma
ICC: Intra-class correlation
i.v.: Intravenous
LDH: Lactate dehydrogenase
MI: Mechanical Index
MIA: Melanoma inhibitory activity
MRI: Magnetic resonance imaging
MTT: Mean transit time
NR: Non-responder
NYHA: New York heart association
PD: Progressive disease
PE: Peak enhancement
PR: Partial remission
RBF: Regional blood flow
RBV: Regional blood volume
RE: Responder
RECIST: Response evaluation criteria in solid tumors
ROI: Region of interest
S100: Protein S100
SD: Stable disease
TTP: Time to peak
UCA: Ultrasound contrast agent
ULN: Upper limit of normal
US: Ultrasound
